# Cost-effectiveness of Nivolumab-Ipilimumab Combination Therapy for the Treatment of Advanced Non–Small Cell Lung Cancer

**DOI:** 10.1001/jamanetworkopen.2021.8787

**Published:** 2021-05-03

**Authors:** P. Travis Courtney, Anthony T. Yip, Daniel R. Cherry, Mia A. Salans, Abhishek Kumar, James D. Murphy

**Affiliations:** 1 University of California, San Diego School of Medicine, La Jolla; 2 Department of Radiation Medicine and Applied Sciences, University of California, San Diego, La Jolla

## Abstract

**Importance:**

Treatment with nivolumab-ipilimumab combination therapy was found to improve overall survival compared with chemotherapy among patients with advanced non–small cell lung cancer (NSCLC) in the CheckMate 227 clinical trial. However, these drugs are substantially more expensive than chemotherapy and, given the high incidence of advanced NSCLC, the incorporation of dual immune checkpoint inhibitors into the standard of care could have substantial economic consequences.

**Objective:**

To assess whether nivolumab-ipilimumab combination therapy is a cost-effective first-line treatment for patients with advanced NSCLC.

**Design, Setting, and Participants:**

This economic evaluation designed a Markov model to compare the cost-effectiveness of nivolumab-ipilimumab combination therapy with platinum-doublet chemotherapy as first-line treatment for patients with advanced NSCLC. The Markov model was created to simulate patients with advanced NSCLC who were receiving either nivolumab-ipilimumab combination therapy or platinum-doublet chemotherapy. Transition probabilities, including disease progression, survival, and treatment toxic effects, were derived using data from the CheckMate 227 clinical trial. Costs and health utilities were obtained from published literature. Data analyses were conducted from November 2019 to September 2020.

**Exposures:**

Nivolumab-ipilimumab combination therapy.

**Main Outcomes and Measures:**

The primary study outcomes were quality-adjusted life-years (QALYs) and cost in 2020 US dollars. Cost-effectiveness was measured using an incremental cost-effectiveness ratio (ICER), with an ICER less than $100 000 per QALY considered cost-effective. Model uncertainty was assessed with 1-way and probabilistic sensitivity analyses.

**Results:**

Treatment with nivolumab-ipilimumab combination therapy was associated with an increase in overall cost of $201 900 and improved effectiveness of 0.50 QALYs compared with chemotherapy, yielding an ICER of $401 700 per QALY. The study model was sensitive to the cost and duration of immunotherapy. Treatment with nivolumab-ipilimumab combination therapy became cost-effective when monthly treatment costs were reduced from $26 425 to $5058 (80.9% reduction) or when the maximum duration of immunotherapy was reduced from 24.0 months to 1.4 months. The model was not sensitive to assumptions about survival or programmed cell death 1 ligand 1 status. A probabilistic sensitivity analysis indicated that, at a willingness-to-pay threshold of $100 000 per QALY, nivolumab-ipilimumab combination therapy was less cost-effective than chemotherapy 99.9% of the time.

**Conclusions and Relevance:**

In this study, first-line treatment with nivolumab-ipilimumab combination therapy was not found to be cost-effective at current prices despite clinical trial data indicating that this regimen increases overall survival among patients with advanced NSCLC.

## Key Points

**Question:**

Is nivolumab-ipilimumab combination therapy cost-effective as first-line treatment for patients with advanced non–small cell lung cancer compared with platinum-doublet chemotherapy?

**Findings:**

In this economic evaluation of the cost-effectiveness of nivolumab-ipilimumab combination therapy, a Markov model was designed to simulate patients with advanced non–small cell lung cancer who were receiving either nivolumab-ipilimumab combination therapy or platinum-doublet chemotherapy. In this model, nivolumab-ipilimumab combination therapy was not found to be cost-effective at a willingness-to-pay threshold of $100 000 per quality-adjusted life-year, with an incremental cost-effectiveness ratio of $401 700 per quality-adjusted life-year compared with chemotherapy.

**Meaning:**

The findings suggest that first-line treatment with nivolumab-ipilimumab combination therapy is not cost-effective at current prices despite clinical trial data indicating that this therapy increases overall survival among patients with advanced non–small cell lung cancer.

## Teaser

This economic evaluation uses data from the CheckMate 227 clinical trial and a Markov model to examine the cost-effectiveness of nivolumab-ipilimumab combination therapy vs platinum-doublet chemotherapy as first-line treatment for patients with advanced non–small cell lung cancer.

## Introduction

Lung cancer represents the second most common type of cancer and the leading cause of cancer-related death in the US,^1^ with non–small cell lung cancer (NSCLC) constituting most of the lung cancer diagnoses. Approximately one-half of patients with NSCLC present with advanced or metastatic disease,^1,2,3^ and a substantial proportion of patients with local or locoregional disease subsequently develop recurrent or metastatic disease.^4,5,6^ Among those with distant disease, the prognosis remains poor, with 5-year relative survival rates of approximately 5%.^1,7^ Given these data, new treatments for this disease are needed.

The treatment options for patients with advanced NSCLC have expanded substantially in recent years. Newer therapies, such as immune checkpoint inhibitors, have increasingly become part of the standard of care for this disease.^8^ The ongoing CheckMate 227 (An Open-Label, Randomized Phase 3 Trial of Nivolumab, or Nivolumab Plus Ipilimumab, or Nivolumab Plus Platinum Doublet Chemotherapy vs Platinum Doublet Chemotherapy in Subjects With Chemotherapy-Naive Stage IV or Recurrent Non–Small Cell Lung Cancer [NSCLC]; [ClinicalTrials.gov Identifier: NCT02477826]) clinical trial recently found that the combination of 2 immune checkpoint inhibitors, nivolumab and ipilimumab, as first-line treatment improved overall survival in patients with advanced NSCLC compared with platinum-doublet chemotherapy, independent of a tumor’s programmed cell death 1 ligand 1 (PD-L1) expression level.^9^ Treatment with nivolumab-ipilimumab combination therapy also improved progression-free survival and had lower rates of adverse events compared with chemotherapy. The clinical trial also included patients who received treatment with nivolumab with or without chemotherapy (depending on PD-L1 expression level) and found nivolumab-ipilimumab combination therapy to be more efficacious than nivolumab monotherapy. In addition, nivolumab-ipilimumab combination therapy resulted in fewer adverse events than nivolumab with chemotherapy but more adverse events than nivolumab without chemotherapy. Overall, these results led to consensus guidelines listing nivolumab-ipilimumab combination therapy as a first-line treatment option for select patients with metastatic NSCLC.^8^

Despite the efficacy of nivolumab-ipilimumab combination therapy for the treatment of advanced NSCLC, one must consider the high costs of these agents. These high costs can have consequences for patients in the form of financial toxicity, leading patients to forgo or delay care, decreasing quality of life, and putting patients at risk of bankruptcy.^10,11,12,13^ Aside from the direct consequences for patients, advanced NSCLC has a high incidence rate, and the widespread adoption of costly drugs could add to the increasing costs of cancer care in general. These economic health care concerns suggest that assessment of the value or cost-effectiveness of these drugs is needed. In this study, we aimed to evaluate the cost-effectiveness of nivolumab-ipilimumab combination therapy compared with chemotherapy as first-line treatment for patients with advanced NSCLC.

## Methods

This economic evaluation followed standard guidelines for the design, analysis, and reporting of our model,^14^ including the Consolidated Health Economic Evaluation Reporting Standards (CHEERS) reporting guideline for economic evaluations. This study included published clinical trial data without individual patient data and was therefore deemed exempt from review by the institutional review board of the University of California, San Diego.

### Decision Model

**Figure 1.**
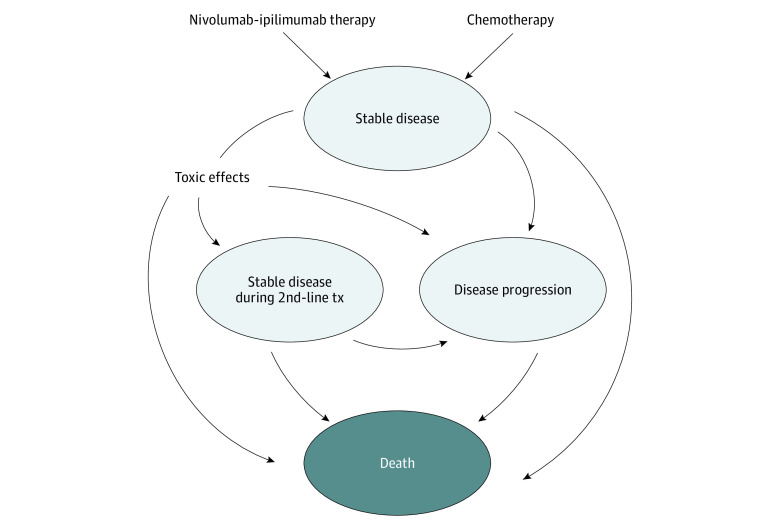
Diagram of Transitions Between Health States Arrows represent transitions between health states. tx indicates treatment.

We constructed a Markov model to simulate costs, quality of life, toxic effects, progression, and survival among patients receiving nivolumab-ipilimumab combination therapy or chemotherapy as first-line treatment for advanced NSCLC. We built our model and derived model inputs based on results reported in the CheckMate 227 clinical trial.^9^ In our model, patients could progress through 4 main health states: stable disease, stable disease during receipt of second-line treatment, disease progression, and death. Figure 1 illustrates the possible transitions between health states. We used a 1-month cycle length and a horizon extending over 10 years. TreeAge Pro Healthcare software, version 2020 R1.2 (TreeAge Software, LLC) was used to construct and analyze our Markov models.

### Treatment Details

The CheckMate 227 clinical trial stratified patients by PD-L1 expression level (≥1% or <1%) and randomized patients to receive chemotherapy alone, nivolumab therapy with or without chemotherapy (depending on PD-L1 level), or nivolumab-ipilimumab combination therapy. Given that the cost-effectiveness of mono–checkpoint inhibition among patients with advanced NSCLC has been evaluated elsewhere,^15,16,17^ we sought to assess the cost-effectiveness of dual checkpoint inhibition with nivolumab-ipilimumab combination therapy compared with chemotherapy. Our primary cost-effectiveness analysis used all patient data from the CheckMate 227 clinical trial (regardless of PD-L1 status). Sensitivity analyses were performed to evaluate cost-effectiveness within the PD-L1 groups (<1% and ≥1%) separately. We also evaluated cost-effectiveness among patients with PD-L1 levels of 50% or higher in a separate sensitivity analysis.

Our base case model followed the CheckMate 227 trial protocol, in which patients received treatment with nivolumab-ipilimumab combination therapy until disease progression, development of unacceptable toxic effects, or 2 years of treatment time, whichever occurred first. Per the CheckMate 227 protocol, certain patients could continue receiving immunotherapy after disease progression if they met prespecified criteria; therefore, we conducted a sensitivity analysis in which patients who experienced disease progression continued to receive immunotherapy for up to 2 years regardless of disease progression. In accordance with the Checkmate 227 protocol, patients who were receiving upfront chemotherapy in our base case model also received treatment until disease progression, development of unacceptable toxic effects, or 3 months (4 chemotherapy cycles) of treatment time, whichever occurred first. In addition, per the CheckMate 227 protocol, patients with nonsquamous histologic characteristics could optionally receive maintenance pemetrexed chemotherapy until disease progression or unacceptable toxic effects. The proportion of patients in the CheckMate 227 clinical trial who received maintenance pemetrexed was not published. Therefore, our base case model did not include maintenance pemetrexed; however, we performed a separate sensitivity analysis in which all patients with nonsquamous histologic characteristics received maintenance pemetrexed. In all treatment arms, we assumed that patients who discontinued first-line treatment transitioned to a second-line systemic treatment (37.7% of patients in the nivolumab-ipilimumab therapy arm and 53.7% of patients in the chemotherapy arm), as reported by the CheckMate 227 clinical trial.

### Model Probabilities

**Figure 2.**
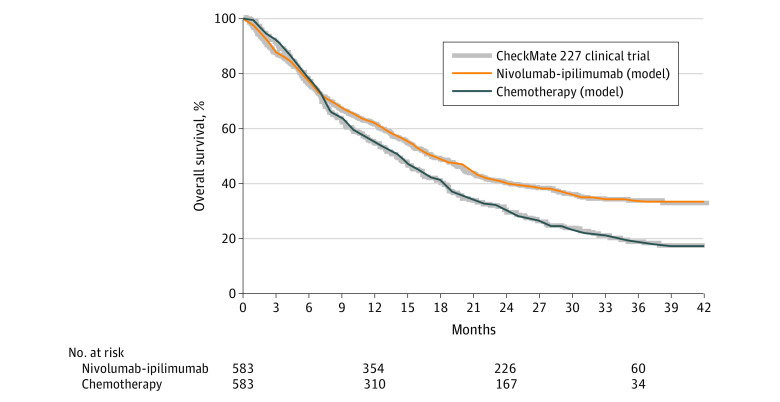
Model Validation Comparison between overall survival curves reported by the CheckMate 227 clinical trial and overall survival estimates produced by the model used in the present study.

Patients entered the Markov model in the stable disease state; they could then remain in the stable disease state or experience toxic effects, disease progression, or death. The transition probabilities for these events were derived from CheckMate 227 data. Similar to previous cost-effectiveness studies,^18,19,20^ we included and evaluated only grades 3 to 5 treatment-related adverse events. Progression and survival data were extracted from the reported Kaplan-Meier curves using previously described methods.^19,21,22^ Model validation is shown in Figure 2 and eTable 1 in the Supplement, with model-predicted survival overlying the Kaplan-Meier estimates from the CheckMate 227 clinical trial. Of note, the CheckMate 227 study reported survival data through 42 months after the initiation of treatment. For survival after 42 months, we considered 2 assumptions. Our base case analysis assumed that all patients who were alive after 42 months would follow conditional survival probabilities of a generalized cohort of patients with advanced NSCLC that were estimated using data from the Surveillance, Epidemiology, and End Results database.^23^ We tested a second scenario using a sensitivity analysis in which we assumed all patients who were alive after 42 months were cured of their cancer and followed the age-adjusted survival probabilities of the general US population provided by actuarial life tables from the US Social Security Administration.^24^

### Costs

We considered costs from both a health care (base case analysis) and societal perspective. Drug costs were calculated by summing the drug’s average wholesale price^25,26,27,28,29,30^ with a 7% reduction^19,22^ plus with the costs of infusion^19^ and follow-up and monitoring.^31^ Details regarding the ways in which drug costs were estimated are available in the eMethods in the Supplement. Toxic effects costs were included as a weighted average based on the number of reported toxic effects in the clinical trial. The costs of grades 3 and 4 toxic effects that were incorporated into our model are summarized in eTable 2 in the Supplement. In addition to formal health care sector costs, our societal perspective model incorporated informal health care costs (patient time and/or salary,^32^ transportation,^33^ and caregiver^34^ costs) and non–health care sector costs (productivity loss^32^). All costs were adjusted to 2020 US dollars using the Consumer Price Index^35^ and are shown in the Table along with their respective literature sources.^25,26,27,28,29,30,32,33,34,36,37,38,39,40,41,42,43,44^

**Table.** Parameters for Base Case Cost-effectiveness Model

Parameter	Value (95% CI), $^a^	Distribution	Source
Drug costs per cycle^b^^,^^c^			
Nivolumab	14 975 (9703-21 417)	Gamma	AWP^25^
Ipilimumab	11 450 (7413-16 296)	Gamma	AWP^26^
Combination nivolumab-ipilimumab	26 425 (17 089-37 662)	Gamma	AWP^25,26^
Pemetrexed	7990 (5182-11 395)	Gamma	AWP^27^
Gemcitabine	118 (76-169)	Gamma	AWP^28^
Cisplatin	94 (61-134)	Gamma	AWP^29^
Carboplatin	163 (105-233)	Gamma	AWP^30^
Chemotherapy total	7929 (5151-11 331)	Gamma	AWP^27,28,29,30^
Second-line treatment in nivolumab-ipilimumab arm^d^	8908 (5764-12 735)	Gamma	AWP^25,26,27,28,29,30,36,37^
Second-line treatment in chemotherapy arm^d^	12 093 (7824-17 255)	Gamma	AWP^25,26,27,28,29,30,36,37^
Drug toxic effects costs^e^			
Nivolumab-ipilimumab	1185 (767-1695)	Gamma	Niraula et al,^38^ 2014; Hornberger et al,^39^ 2015; Smith et al,^40^ 2002; Insinga et al,^41^ 2019
Chemotherapy	6384 (4139-9127)	Gamma	Niraula et al,^38^ 2014; Hornberger et al,^39^ 2015; Smith et al,^40^ 2002; Insinga et al,^41^ 2019
Disease costs per cycle			
Stable disease	2166 (1397-3098)	Gamma	Insinga et al,^41^ 2019
Progressed disease	4000 (2575-5712)	Gamma	Insinga et al,^41^ 2019
Palliative care and death (1-time cost)	15 957 (10 335-22 818)	Gamma	Insinga et al,^41^ 2019
Societal costs per cycle			
Patient time and salary loss	534 (345-763)	Gamma	Guerin et al,^32^ 2016
Parking, meals, and travel in nivolumab-ipilimumab arm	91 (59-130)	Gamma	Lauzier et al,^33^ 2011
Parking, meals, and travel in chemotherapy arm	61 (39-87)	Gamma	Lauzier et al,^33^ 2011
Caregiver	619 (401-882)	Gamma	Li et al,^34^ 2013
Productivity loss	854 (553-1219)	Gamma	Guerin et al,^32^ 2016
Health utilities			
Disease status utility per y			
Stable disease	0.754 (0.407-0.970)	Beta	Nafees et al,^42^ 2017
Disease progression (decrement)	0.180 (0.115-0.367)	Beta	Nafees et al,^43^ 2008
Drug toxic effects disutility^f^			
Nivolumab-ipilimumab	0.017 (0.011-0.024)	Beta	Hornberger et al,^39^ 2015; Freeman et al,^44^ 2015; Nafees et al,^42^ 2017
Chemotherapy	0.019 (0.012-0.027)	Beta	Hornberger et al,^39^ 2015; Freeman et al,^44^ 2015; Nafees et al,^42^ 2017
Death	0	NA	NA

Abbreviations: AWP, average wholesale price; NA, not applicable.

^a^ Costs are in 2020 US dollars and adjusted for inflation as appropriate.

^b^ Includes costs of drug infusion ($143)^19^ and follow-up and monitoring ($433).^31^

^c^ Average wholesale price with 7% reduction.

^d^ Calculated as the average cost of treatment using weighted frequencies of individual second-line therapeutic agents received by each treatment arm in the CheckMate 227 clinical trial.

^e^ Calculated as the average cost of toxic effects using weighted frequencies of grade 3 to 4 treatment-related adverse events for each treatment arm in the CheckMate 227 clinical trial. Costs of individual toxic effects were derived from the literature and include all care required to manage each toxic effect. References for individual toxic effect costs are summarized in eTable 2 in the Supplement.

^f^ Calculated as the average disutility of toxic effects using weighted frequencies of grade 3 to 4 treatment-related adverse events for each treatment arm in the CheckMate 227 clinical trial. Disutility from experiencing toxic effects occurred over a 1-month period. Disutilities of individual toxic effects were derived from the literature. References for individual toxic effect disutilities are summarized in eTable 3 in the Supplement.

### Outcome Measures

Treatment effectiveness was measured in quality-adjusted life-years (QALYs), which is a weighted aggregate of health utilities over time. Health utility is measured on a scale of 0 to 1, with 1 corresponding to optimal health and 0 corresponding to death^14^; specific values in this study were obtained from published literature. Experiencing toxic effects was considered a decrement in health utility (otherwise known as a disutility) that extended over a 1-month period. Similar to toxic effects costs, we calculated weighted averages for the disutility associated with specific toxic effect events that paralleled the frequency of events in the CheckMate 227 clinical trial (eTable 3 in the Supplement). Health utility values are summarized in the Table along with their respective literature sources.^39,42,43,44^ An annual discount rate of 3% was applied to all costs and QALYs.

### Statistical Analysis

Cost-effectiveness was measured using an incremental cost-effectiveness ratio (ICER), which is the ratio of the differences in cost (measured in US dollars) and effectiveness (measured in QALYs) between the 2 treatments. We used a willingness-to-pay threshold of $100 000 per QALY,^45^ with ICERs less than $100 000 per QALY considered cost-effective.^19,21,22,45,46^ The ICERs were rounded to the nearest $100. We performed 1-way deterministic sensitivity analyses of each variable in the model to evaluate which variables had the greatest consequences for cost-effectiveness. To further assess model uncertainty, we performed a probabilistic sensitivity analysis using a Monte Carlo simulation with 100 000 repetitions, allowing us to simultaneously vary uncertainty in cost, health utilities, and transition probabilities. In our probabilistic sensitivity analysis, cost variables were modeled with gamma distributions, and health utilities and transition probabilities were modeled with beta distributions. Standard deviations for each distribution were obtained from the literature, when possible. Unknown SDs were calculated using 20% of the mean.^19,47^ We also tested a range of possible unknown SDs (10%-40% of the mean), which did not change our results. Data analyses were conducted from November 2019 to September 2020.

## Results

### Base Case Analysis

Our base case analysis indicated that nivolumab-ipilimumab combination therapy was associated with a $201 900 increase in the overall cost of treatment from $175 500 for chemotherapy to $377 400 for nivolumab-ipilimumab therapy. Treatment with nivolumab-ipilimumab combination therapy was associated with an increase in effectiveness of 0.50 QALYs from 1.18 QALYs for chemotherapy to 1.68 QALYs for nivolumab-ipilimumab combination therapy. This increase yielded an ICER of $401 700 per QALY, which would not be considered cost-effective at a willingness-to-pay threshold of $100 000 per QALY. Considering cost-effectiveness from a societal perspective yielded an ICER of $434 400 for nivolumab-ipilimumab combination therapy compared with chemotherapy.

### One-Way Sensitivity Analyses

**Figure 3.**
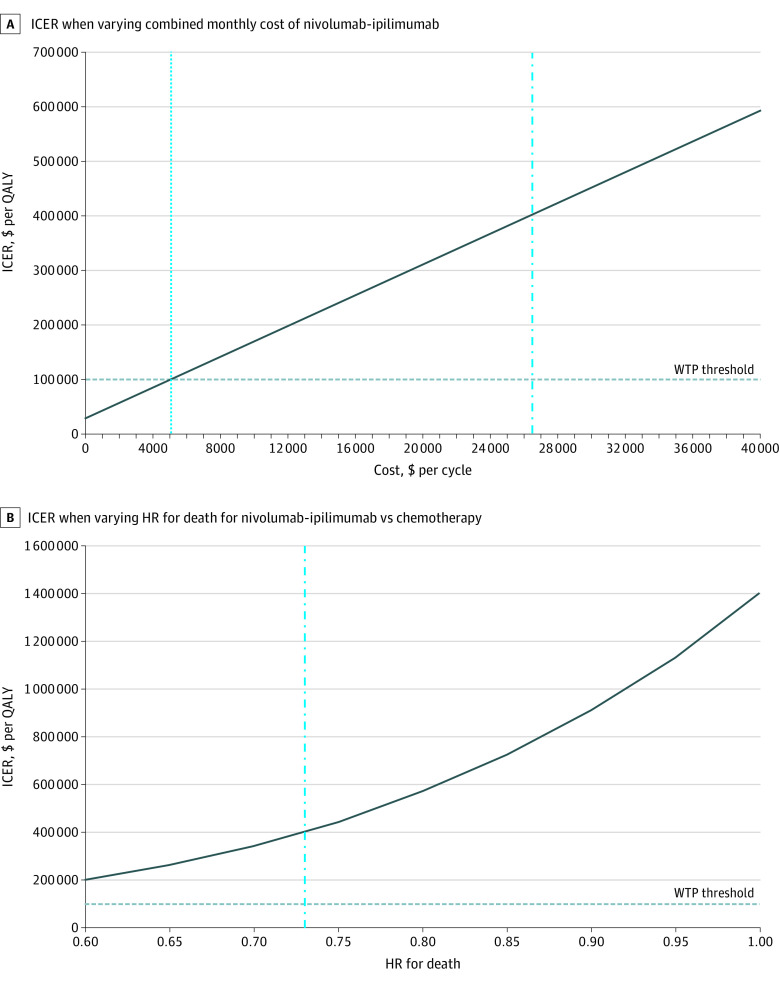
One-Way Sensitivity Analyses Graphs represent the incremental cost-effectiveness ratios (ICERs) of combined nivolumab-ipilimumab therapy compared with chemotherapy. A, The combined monthly cost of nivolumab-ipilimumab therapy would have to decrease from $26 425 (base case, represented by the vertical dashed line) to $5058 (represented by the vertical dotted line) to become cost-effective compared with chemotherapy at a willingness to pay (WTP) threshold of $100 000 per quality-adjusted life-year (QALY). B, The ICERs below the WTP threshold of $100 000 per QALY represent scenarios in which nivolumab-ipilimumab therapy would be considered cost-effective compared with chemotherapy. The vertical dashed line represents the base case hazard ratio (HR) of 0.73.

In the 1-way sensitivity analyses, our model was modestly sensitive to the cost and duration of nivolumab-ipilimumab combination therapy (eFigure in the Supplement). The monthly combined cost of nivolumab-ipilimumab combination therapy would need to decrease from $26 425 to $5058 (an 80.9% reduction) to become cost-effective at a willingness-to-pay threshold of $100 000 per QALY (Figure 3A). Our base case analysis assumed that patients received nivolumab-ipilimumab combination therapy until disease progression, unacceptable toxic effects, or 24 months of follow-up. The maximum allowable duration of nivolumab-ipilimumab treatment would have to decrease from 24.0 months to 1.4 months to become cost-effective at a willingness-to-pay threshold of $100 000 per QALY. The CheckMate 227 trial protocol allowed patients to receive nivolumab-ipilimumab combination therapy beyond disease progression if they met prespecified criteria. When we adjusted our model so that all patients receiving nivolumab-ipilimumab continued to receive combination immunotherapy beyond disease progression and for up to 24 months, the ICER of nivolumab-ipilimumab therapy increased to $551 900 per QALY. When we adjusted our model so that patients with nonsquamous histologic characteristics who were receiving chemotherapy continued to receive maintenance pemetrexed chemotherapy, the ICER decreased slightly to $363 400 per QALY.

The cost-effectiveness model was not sensitive to other costs, health utilities, assumptions about toxic effects, or survival. For example, the CheckMate 227 clinical trial found a hazard ratio (HR) of 0.73 (95% CI, 0.64-0.84) for the risk of death among patients receiving nivolumab-ipilimumab combination therapy compared with chemotherapy. When we assumed an HR of 0.64 (the lower end of the 95% CI) for the risk of death, the ICER decreased to $249 300 per QALY (Figure 3B). Our base case model assumed that survival beyond the CheckMate 227 clinical trial range would be consistent with data from the Surveillance, Epidemiology, and End Results database regarding patients with advanced NSCLC. When we assumed that all patients alive at the end of the study (42 months) were cured of disease, the ICER decreased to $317 300 per QALY.

Our model was also not particularly sensitive to PD-L1 expression level. When we incorporated disease progression, survival, and toxic effects data for the cohorts of patients with PD-L1 levels less than 1%, 1% or higher, and 50% or higher, we found ICERs of $332 100 per QALY, $440 100 per QALY, and $375 700 per QALY, respectively. The results of all sensitivity analyses are summarized in eTable 4 in the Supplement.

### Probabilistic Sensitivity Analysis

**Figure 4.**
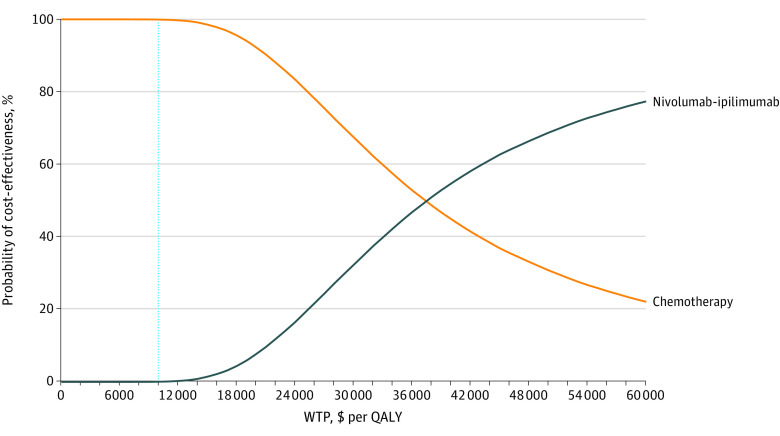
Cost-effectiveness Acceptability Curve Results of the probabilistic sensitivity analysis comparing cost-effectiveness of nivolumab-ipilimumab combination therapy with chemotherapy, with a willingness to pay (WTP) threshold of $100 000 per quality-adjusted life-year (QALY).

Probabilistic sensitivity analysis comparing the cost-effectiveness of nivolumab-ipilimumab combination therapy vs chemotherapy found that, at a willingness-to-pay threshold of $100 000 per QALY, chemotherapy would be the cost-effective option 99.9% of the time (Figure 4). When we increased the willingness-to-pay threshold to $200 000 per QALY, chemotherapy would remain the cost-effective option 92.2% of the time.

## Discussion

In this cost-effectiveness study, we found that nivolumab-ipilimumab combination therapy compared with chemotherapy could not be considered cost-effective as first-line treatment for patients with advanced NSCLC. Our model was not particularly sensitive to assumptions regarding costs, health utilities, or transition probabilities. Of note, the monthly price of nivolumab-ipilimumab combination therapy would have to be reduced from $26 425 to $5058 (an 80.9% reduction) to be cost-effective compared with chemotherapy. In addition, our model found that the maximum duration of treatment with nivolumab-ipilimumab combination therapy would need to decrease to 1.4 months to become cost-effective.

The question of immunotherapy duration remains unanswered in oncologic research; however, one study of patients with advanced melanoma found that those who received treatment with pembrolizumab-nivolumab combination therapy for less than 6 months had higher risks of disease progression compared with those who received treatment for more than 6 months.^48^ The CheckMate 153 (A Safety Trial of Nivolumab in Patients With Advanced or Metastatic Non–Small Cell Lung Cancer Who Have Progressed During or After Receiving At Least 1 Prior Chemotherapy Regimen [ClinicalTrials.gov Identifier: NCT02066636]) clinical trial also found that patients with advanced NSCLC who discontinued nivolumab after 1 year had worse progression-free survival compared with patients who received continuous treatment.^49^ The ongoing DICIPLE (Double Immune Checkpoint Inhibitors in PD-L1–Positive Stage IV Non–Small Cell Lung Cancer [ClinicalTrials.gov Identifier: NCT03469960]) phase 3 clinical trial of patients with advanced NSCLC will further assess the consequences of nivolumab-ipilimumab treatment duration; however, it seems unlikely that the duration required for optimal impact will decrease to the point at which dual checkpoint inhibition would become cost-effective. Of note, our model was not particularly sensitive to assumptions about survival, and cost-effectiveness did not depend on patients’ PD-L1 expression level.

To our knowledge, 2 studies exist on the cost-effectiveness of nivolumab-ipilimumab combination therapy for the treatment of advanced NSCLC.^50,51^ The first study^50^ reported slightly lower ICERs when comparing nivolumab-ipilimumab combination therapy with chemotherapy ($143 434 per QALY vs $180 307 per QALY, respectively). The researchers used a higher willingness-to-pay threshold ($150 000 per QALY) and concluded that nivolumab-ipilimumab combination therapy could be considered cost-effective among patients with a PD-L1 level of less than 1%. The second study^51^ also found slightly lower ICERs when comparing nivolumab-ipilimumab combination therapy with chemotherapy ($107 404 per QALY vs $172 589 per QALY, respectively) and used a willingness-to-pay threshold of $150 000 per QALY. In contrast to the first study, researchers in the second study concluded that nivolumab-ipilimumab combination therapy could be considered cost-effective among patients with PD-L1 levels of 50% or higher and 1% or higher but not among patients with PD-L1 levels of less than 1%. Our study found both higher incremental costs and lower incremental effectiveness (as measured by QALY) for nivolumab-ipilimumab combination therapy compared with these other studies, which produced substantially higher ICERs in our analysis.

Our methodology differs from the 2 previous cost-effectiveness studies in several areas. First, we used a different approach to model disease progression and survival that used Kaplan-Meier estimates in the CheckMate 227 clinical trial and incorporated long-term poststudy survival estimates from different outside resources. Second, our approach to modeling health utility for stable disease and disease progression differed from those studies. Overall, our model was not particularly sensitive to assumptions about survival or health utilities, which suggests that these factors individually would not account for differences between the results of our study and those of other studies that reported lower ICERs. However, a series of small differences in model construction and variable choice may explain the differences in ICERs between our study and previous research.

When considering the cost-effectiveness of nivolumab-ipilimumab combination therapy for the treatment of other cancer types, the regimen was found to be cost-effective for patients with advanced renal cell carcinoma depending on the willingness-to-pay threshold used.^20,31,52^ However, for the treatment of advanced melanoma^53,54,55^ and metastatic colorectal cancer,^56^ nivolumab-ipilimumab combination therapy was not found to be cost-effective compared with a variety of other treatments, including both immunotherapy and chemotherapy. Several cost-effectiveness studies^57,58,59,60,61,62,63^ have investigated nivolumab monotherapy as second-line treatment for patients with advanced NSCLC. Many studies have compared nivolumab monotherapy with docetaxel monotherapy, and most of those studies have found that nivolumab was not cost-effective compared with docetaxel in a variety of health care settings.^57,58,59,60,61,62^ In addition, nivolumab was not found to be cost-effective compared with erlotinib^57^ and nivolumab was dominated by atezolizumab (ie, less effective and more expensive than atezolizumab).^63^ However, the results of 2 other studies have suggested that introducing PD-L1 testing or basing treatment on different PD-L1 expression thresholds may improve the cost-effectiveness of nivolumab.^58,59^ Overall, the results of the current study support the consensus that nivolumab and/or ipilimumab therapies cannot be considered cost-effective when compared with standard, less expensive therapies.

This study raises several points regarding the implications of the high costs associated with immunotherapy for the treatment of cancer.^17,64,65^ Among patients with lung cancer in particular, a substantial number of individuals present with metastatic disease or develop recurrent disease after initial treatment of local or locoregional disease.^3,4,5,6^ Thus, a large number of patients with NSCLC will be affected by these high drug costs. Nivolumab-ipilimumab combination therapy was recently approved by the US Food and Drug Administration for use as first-line treatment among patients who have metastatic NSCLC and PD-L1 levels of 1% or higher^66^ or as treatment in combination with chemotherapy for patients with metastatic NSCLC regardless of PD-L1 status.^67^ However, Food and Drug Administration approval of a treatment does not guarantee insurance coverage.^68^ Most insurers provide some coverage for immune checkpoint inhibitors^69,70^; however, even with insurance coverage, the high baseline costs of these drugs can lead to high out-of-pocket costs for patients regardless of individual insurance plans.^13^ Observational research has found an association between the high costs of cancer care and decreased patient adherence, diminished quality of life, and increased risk of substantial debt or bankruptcy.^10,11,12,13^ Overall, the impact of cost represents an important yet underappreciated and understudied factor associated with the health outcomes of patients with cancer.

### Limitations

This study has several limitations. Our model construction primarily used data from a single randomized clinical trial. Cost-effectiveness research would ideally draw from a wider array of resources to construct a more robust predictive model. Treatment responses, quality of life, and costs vary substantially by individual patient, and while our societal viewpoint analysis indicated that nivolumab-ipilimumab combination therapy was not cost-effective, the results may differ for individual patients or for health care delivery settings outside of the CheckMate 227 clinical trial.

Another potential limitation is the heterogeneous array of resources used to inform estimations about costs and health utilities. However, our model was not particularly sensitive to assumptions about cost or health utility, which suggests that including more accurate estimations would be unlikely to change our results. Of note, our analysis considered only dual checkpoint inhibition using nivolumab-ipilimumab combination therapy compared with chemotherapy. Many alternative treatment options (including nivolumab monotherapy and other systemic therapies or immunotherapies) exist for the treatment of advanced NSCLC.^8^ The addition of more comparator treatment arms to this study could, in theory, change the cost-effectiveness of nivolumab-ipilimumab combination therapy; however, the ICER of nivolumab-ipilimumab combination therapy was substantially outside the range considered cost-effective, which suggests that adding more treatment arms would be unlikely to alter our conclusions.

## Conclusions

This economic evaluation found that nivolumab-ipilimumab combination therapy could not be considered cost-effective as first-line treatment for patients with advanced NSCLC compared with standard chemotherapy despite the increases in overall survival among patients receiving nivolumab-ipilimumab combination therapy. Although immunotherapy represents a promising area in cancer treatment, one must consider the consequences of its high costs to provide the best patient care.

## Notes

Supplement. **eMethods.** Methods for Determining Drug Costs **eFigure.** Tornado Diagram of 1-Way Sensitivity Analyses **eTable 1.** Model Validation **eTable 2.** Associated Costs of Grade 3 to 4 Treatment-Related Adverse Events **eTable 3.** Disutility From Grade 3 to 4 Treatment-Related Adverse Events **eTable 4.** Results of 1-Way Sensitivity Analysis **eReferences**
