# Errors in x-Axis Labeling in Figure

**DOI:** 10.1001/jamanetworkopen.2021.18586

**Published:** 2021-06-16

In the Original Investigation titled “Cost-effectiveness of Nivolumab-Ipilimumab Combination Therapy for the Treatment of Advanced Non–Small Cell Lung Cancer,”^1^ published May 3, 2021, there were errors in the x-axis tick labels in Figure 4. All of the labels were off by a factor of 10; for example, “6000” should have appeared as “60 000” and so forth along the entire x-axis. This article has been corrected.^1^
