# Supplementary material for: Cost-effectiveness of Nivolumab-Ipilimumab Combination Therapy for the Treatment of Advanced Non–Small Cell Lung Cancer
Source: JAMA Netw Open. 2021 May 3;4(5):e218787. doi: 10.1001/jamanetworkopen.2021.8787 (PMC8094011; doi:10.1001/jamanetworkopen.2021.8787)
Supplement: Supplement. — eMethods. Methods for Determining Drug Costs eFigure. Tornado Diagram of 1-Way Sensitivity Analyses eTable 1. Model Validation eTable 2. Associated Costs of Grade 3 to 4 Treatment-Related Adverse Events eTable 3. Disutility From Grade 3 to 4 Treatment-Related Adverse Events eTable 4. Results of 1-Way Sensitivity Analysis eReferences [file jamanetwopen-e218787-s001.pdf]

Supplementary Online Content

Courtney PT, Yip AT, Cherry DR, Salans MA, Kumar A, Murphy JD. Cost-effectiveness of nivolumab-ipilimumab combination therapy for the treatment of advanced non–small cell lung cancer. *JAMA Netw Open*. 2021;4(5):e218787. doi:10.1001/jamanetworkopen.2021.8787

eMethods. Methods for Determining Drug Costs

eFigure. Tornado Diagram of 1-Way Sensitivity Analyses

eTable 1. Model Validation

eTable 2. Associated Costs of Grade 3 to 4 Treatment-Related Adverse Events

eTable 3. Disutility From Grade 3 to 4 Treatment-Related Adverse Events

eTable 4. Results of 1-Way Sensitivity Analysis

eReferences

This supplementary material has been provided by the authors to give readers additional information about their work.

© 2021 Courtney PT et al. *JAMA Network Open*.

eMethods. Methods for Determining Drug Costs

Per the CheckMate 227 trial, patients in the nivolumab-ipilimumab arm received nivolumab at a dose of 3 mg/kg body weight every two weeks and ipilimumab at a dose of 1 mg/kg body weight every 6 weeks. In the chemotherapy arm, patients received platinum-doublet chemotherapy every three weeks for up to four cycles. The specific chemotherapeutic agents and dosing used in CheckMate 227 varied by tumor histology (squamous versus nonsquamous); therefore, with our costs of chemotherapy, we calculated a weighted average between the two tumor histologic types reported in the trial. With drug cost calculations, we used a body weight of 73kg, based on the male-female proportions in CheckMate 227 using sex-specific body weights[1], and a body surface area of 1.78m^2, based on cancer patient-specific averages[2]. In the sensitivity analysis including maintenance pemetrexed for chemotherapy patients, the cost of maintenance pemetrexed was based on the CheckMate 227 trial's specified dose (500mg/m^2), the proportion of patients with nonsquamous histology, and was assumed to be given every three weeks. Second-line treatment costs[36,37] were calculated as frequency-weighted averages using reported subsequent treatments received by at least 3% of patients for each treatment arm in CheckMate 227. We did not include "targeted therapy" in second-line treatment costs in either arm because those agents were not specified. Drug costs were adjusted to a monthly rate to match the cycle length of our model. Costs of toxicities[38-41], stable disease[41], progression[41], and death[41] were obtained from the literature.

© 2021 Courtney PT et al. *JAMA Network Open*.

eFigure. Tornado Diagram of 1-Way Sensitivity Analyses

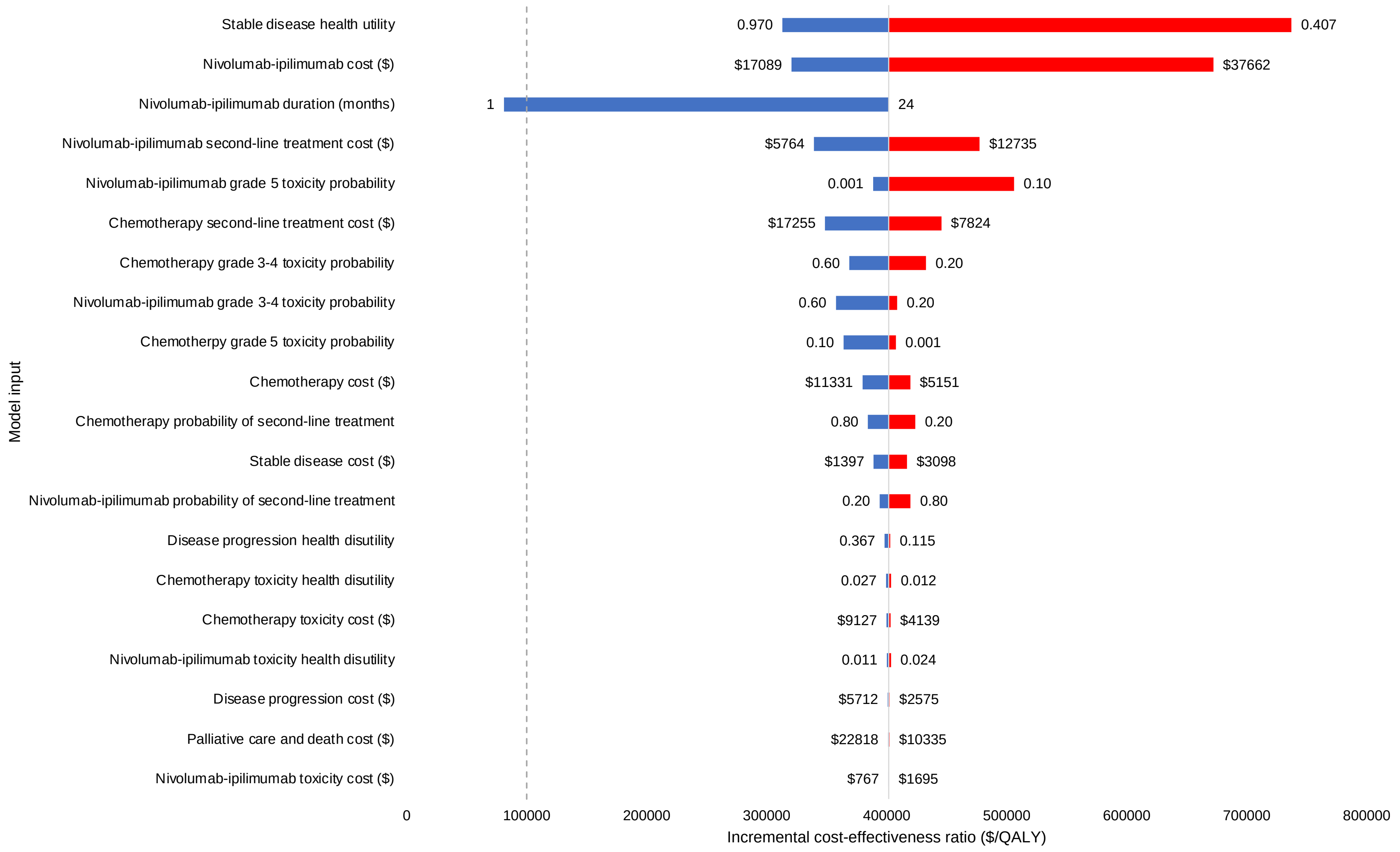

This graph represents the incremental cost-effectiveness ratio (ICER) of nivolumab-ipilimumab compared to chemotherapy in all patients when individually varying the parameters of the base case cost-effectiveness model. The solid vertical line represents the base case analysis ICER ($401,700/QALY) for nivolumab-ipilimumab compared to chemotherapy, and the vertical dashed line represents the willingness-to-pay threshold of $100,000/QALY.

© 2021 Courtney PT et al. *JAMA Network Open*.

eTable 1. Model Validation

Study End Point	Cost-Effectiveness Model, %	CheckMate 227 Trial, %
Overall Survival (2 year)		
Nivolumab + ipilimumab	40.2	40
Chemotherapy	30.2	30
Progression-Free Survival (2 year)		
Nivolumab + ipilimumab	20.1	20
Chemotherapy	6.4	6
Grade 3-4 Toxicity		
Nivolumab + ipilimumab	32.8	32.8
Chemotherapy	36.0	36.0
Grade 5 Toxicity		
Nivolumab + ipilimumab	1.4	1.4
Chemotherapy	1.1	1.1

This table quantitively compares 2-year overall and progression-free survival rates and grade 3-4 and grade 5 treatment-related toxicities reported in the CheckMate 227 trial with those produced by our model.

© 2021 Courtney PT et al. *JAMA Network Open*.

eTable 2. Associated Costs of Grade 3 to 4 Treatment-Related Adverse Events

Adverse Event[a]	No. of patients (%)[b]	Costs in 2020 USD[c]	Reference
**Nivolumab-ipilimumab**			
Fatigue, asthenia	18 (3.1)	1,065.44	Niraula et al[38], 2014
Rash, pruritus	12 (2.1)	272.33	Hornberger et al[39], 2015
Diarrhea	10 (1.7)	169.93	Hornberger et al[39], 2015
Decreased appetite, nausea, vomiting	9 (1.6)	160.13	Hornberger et al[39], 2015
Anemia	8 (1.4)	5,243.47	Smith et al[40], 2002
Weighted average[d]	-	*1,184.81*	
**Chemotherapy**			
Anemia	66 (11.6)	5,243.47	Smith et al[40], 2002
Neutropenia	54 (9.5)	16,857.15	Hornberger et al[39], 2015
Neutrophil count decreased	36 (6.3)	907.00	Insinga[41], 2019
Decreased appetite, nausea, vomiting	32 (5.6)	160.13	Hornberger et al[39], 2015
Fatigue, asthenia	13 (2.2)	1,065.44	Niraula et al[38], 2014
Diarrhea	4 (0.7)	169.93	Hornberger et al[39], 2015
Weighted average[d]	-	*6,383.72*	

[a]Refers to treatment-related adverse events of any grade that occurred in ≥15% of total patients in the CheckMate 227 trial. Our analysis only included and evaluated grade 3-4 treatment-related adverse events.
[b]Number within treatment arm: nivolumab-ipilimumab (N=576), chemotherapy (N=570).
[c]Cost per one-month cycle.
[d]Calculated as an average cost of toxicity using the weighted frequency of occurrence. This value was used in the base-case model.

© 2021 Courtney PT et al. *JAMA Network Open*.

eTable 3. Disutility From Grade 3 to 4 Treatment-Related Adverse Events

Adverse Event[a]	Number of patients (%)[b]	Disutility[c]	Reference
**Nivolumab-ipilimumab**			
Fatigue, asthenia	18 (3.1)	0.024	Nafees et al[43], 2017
Rash, pruritus	12 (2.1)	0.013	Nafees et al[43], 2017
Diarrhea	10 (1.7)	0.018	Nafees et al[43], 2017
Decreased appetite, nausea, vomiting	9 (1.6)	0.017	Nafees et al[43], 2017
Anemia	8 (1.4)	0.006	Freeman et al[42], 2015
Weighted average[d]	-	*0.017*	
**Chemotherapy**			
Anemia	66 (11.6)	0.006	Freeman et al[42], 2015
Neutropenia	54 (9.5)	0.029	Nafees et al[43], 2017
Neutrophil count decreased	36 (6.3)	0.029	Hornberger et al[39], 2015
Decreased appetite, nausea, vomiting	32 (5.6)	0.017	Nafees et al[43], 2017
Fatigue, asthenia	13 (2.2)	0.024	Nafees et al[43], 2017
Diarrhea	4 (0.7)	0.018	Nafees et al[43], 2017
Weighted average[d]	-	*0.019*	

[a]Refers to treatment-related adverse events of any grade that occurred in ≥15% of total patients in the CheckMate 227 trial. Our analysis only included and evaluated grade 3-4 treatment-related adverse events.
[b]Number within treatment arm: nivolumab-ipilimumab (N=576), chemotherapy (N=570).
[c]Disutility per one-month cycle.
[d]Calculated as an average disutility of toxicity using the weighted frequency of occurrence. This value was used in the base-case model.

© 2021 Courtney PT et al. *JAMA Network Open*.

eTable 4. Results of 1-Way Sensitivity Analysis

**Model**	**ICER ($/QALY)**
Base Case	401,700
**Perspective**	
Health Care Payer	401,700
Societal	434,400
**Duration of nivolumab-ipilimumab treatment**	
24 months maximum	401,700
12 months maximum	361,700
4 months maximum[a]	235,200
Continue after disease progression[b]	551,900
Continue after grade 3-4 treatment-related adverse event	467,300
**Including maintenance pemetrexed in chemotherapy arm**	363,400
**Survival Assumptions**	
Reduced risk of death from nivolumab-ipilimumab	
27% reduction in risk of death (HR 0.73)[c]	401,700
36% reduction in risk of death (HR 0.64)[d]	249,300
Survival beyond trial range[e]	
All patients alive at 42 months follow SEER survival data for advanced NSCLC	401,700
All patients alive at 42 months follow cured[f] of disease	317,300
Nivolumab-ipilimumab patients alive at 42 months cured[f] of disease[g]	287,800
**PD-L1 Expression Level**	
All patients	401,700
≥1%	440,100
≥50%	375,700
<1%	332,100

Abbreviations: QALY, quality-adjusted life-year; HR, hazard ratio; SEER, Surveillance, Epidemiology, and End Results; NSCLC, non-small cell lung cancer; PD-L1, programmed death-ligand 1.
[a]Median duration of nivolumab-ipilimumab therapy was 4.2 months in CheckMate 227 trial.
[b]Patients remain on nivolumab-ipilimumab for two years per CheckMate 227 protocol.
[c]HR of death in nivolumab-ipilimumab arm compared to chemotherapy in all patients in CheckMate 227 trial. This value was used in base-case model.
[d]Lower end of 95% confidence interval for HR of death in nivolumab-ipilimumab arm compared to chemotherapy in all patients in CheckMate 227 trial.
[e]CheckMate 227 reported survival data through 42 months.
[f]Survival beyond trial range followed US Social Security Administration Actuarial Life Tables[24].
[g]Those on chemotherapy assumed to not be cured of disease, and survival beyond trial range followed SEER data.

© 2021 Courtney PT et al. *JAMA Network Open*.

eReferences

1. Baracos VE, Reiman T, Mourtzakis M, Gioulbasanis I, Antoun S. Body composition in patients with non-small cell lung cancer: a contemporary view of cancer cachexia with the use of computed tomography image analysis. *Am J Clin Nutr*. 2010;91(4):1133S-1137S.

2. Sacco JJ, Botten J, Macbeth F, Bagust A, Clark P. The average body surface area of adult cancer patients in the UK: a multicentre retrospective study. *PLoS One*. 2010;5(1):e8933.

© 2021 Courtney PT et al. *JAMA Network Open*.
